# Effects of a transitional palliative care model on patients with end-stage heart failure: study protocol for a randomized controlled trial

**DOI:** 10.1186/s13063-016-1303-7

**Published:** 2016-03-31

**Authors:** Alina Yee Man Ng, Frances Kam Yuet Wong, Paul Hong Lee

**Affiliations:** School of Nursing, The Hong Kong Polytechnic University, Hung Hom, Kowloon, Hong Kong, China

**Keywords:** Transitional care, Palliative care, End-stage heart failure, Health services utilization, Quality of life

## Abstract

**Background:**

Heart failure (HF) is characterized by high rates of readmission after hospitalization, and readmission is a major contributor to healthcare costs. The transitional care model has proven efficacy in reducing the readmission rate and economic outcomes, and increasing satisfaction with care. However, the effectiveness of the transitional care model has not been evaluated in patients with end-stage HF. This study was designed to compare the customary hospital-based care and a comprehensive transitional care model, namely the Home-based Palliative HF Program (HPHP), in terms of readmission rate, quality of life, and satisfaction with care among end-stage HF patients under palliative care.

**Methods/design:**

This is a randomized controlled trial taking place in hospitals in Hong Kong. We have been recruiting patients with end-stage HF who are identified as appropriate for palliative care during hospitalization, on referral by their physicians. A set of questionnaires is collected from each participant upon discharge. Participants are randomized to receive usual care (customary hospital-based care) or the intervention (HPHP). The HPHP will be implemented for up to 12 months. Outcome measures will be performed at 1, 3, 6, and 12 months post-discharge. The primary outcome of this study is quality of life measured by the Chronic Heart Failure Questionnaire - Chinese version; secondary outcomes include readmission rate, symptom intensity, functional status, and satisfaction with care.

**Discussion:**

This study is original and will provide important information for service development in the area of palliative care. The introduction of palliative care to end-stage organ failure patients is new and has received increasing attention worldwide in the last decade. This study adopts the randomized controlled trial, a vigorous research design, to establish scientific evidence in exploring the best model for end-stage HF patients receiving palliative care.

**Trial registration:**

This trial was registered as NCT02086305 on 7 March 2014 in the United States Clinical Trials Registration, and in the Clinical Trials Registry, Hong Kong University with the trial number UW12202.

**Electronic supplementary material:**

The online version of this article (doi:10.1186/s13063-016-1303-7) contains supplementary material, which is available to authorized users.

## Background

Heart failure (HF) is a global health issue affecting more than 5 million people in the United States (US) [1] and 14 million Europeans [2]. In Hong Kong, the overall HF ratio per 1,000 people is 0.7, resulting in a total of over 5,000 cases; the new:old case ratio is 2.8:1 [3]. Heart failure is the leading cause of death in the US, accounting for 31.4 % of all deaths and followed by cancer and stroke [4]. In Hong Kong, cancer is still the leading cause of death (30.6 %), but heart disease was ranked second (15.5 %) among the 42,705 deaths in 2009 [5]. The management of HF in recent years has improved, but the mortality rate remains high, with a reported 40 % death rate within the first year after diagnosis [6, 7] and 75 % at five years [8]. Patients with heart failure tend to need more emergency room visits, repeated hospital admissions, and longer lengths of stay [1]. In Hong Kong, around 25–50 % of HF patients are readmitted within 6 months of discharge [9]. The repeated use of services results in increased healthcare expenditures [10, 11], costing US$30 billion a year in the US [1] and GBP 905 million in the United Kingdom [8].

The prognosis for patients with HF is poor, and various burdens become prominent when HF progresses to the end stage. End-stage heart failure is resistant to medical therapy [1], and this group of patients experiences a marked reduction in health-related quality of life [6, 12], encountering health problems encompassing physical, psychological, social, and spiritual aspects. Topping the list of physical symptoms are fatigue, dyspnea, and edema [6, 12, 13]. Other reported symptoms include decreased appetite, cough, dry mouth, and pain [6, 12]. With their progressive decline in functional status, these patients often have increasing difficulty dealing with daily activities [14, 15]. Psychologically, some patients with HF live in the shadow of fear [16], experiencing emotional turbulence [17] and feeling frustrated with their sense of personal failure [18]. Patients show symptoms of insomnia, worry, sadness, and irritability [6, 12]. Socially, patients with HF feel isolated and lonely [14, 19–21], often relying on others for daily living assistance and thus regarding themselves as a burden to their carers [19]. These patients also face spiritual issues, such as questioning the meaning of living with severe HF as they experience strong feelings of uncertainty and hopelessness [20]. In fact, patients understand that their condition could change rapidly, including a sudden decline resulting in death [20, 22]. Apart from the symptom burden, heart failure also imposes a financial burden on the healthcare system, particularly in the form of hospital readmission [23, 24]. Heart failure is a leading cause of hospitalization, and a systematic review confirmed that about 25 % of patients hospitalized with HF are readmitted within 30 days [23]. Reasons for hospital readmission were worsening of symptoms, unavoidable progression of illness, distressing psychosocial issues, inadequate self-care adherence, and lack of knowledge on how to seek help from the healthcare provider [24, 25]. Therefore, it is essential to address end-stage HF patients’ physical, psychological, and spiritual needs in order to facilitate a better hospital-to-home transition for them.

Patients with heart failure suffer from a number of symptoms that profoundly impact their quality of life (QoL) towards the end of life [1, 26]. They share similar symptoms with patients who have cancer, including breathlessness, fatigue, and edema [27–29]. QoL concerns are also shared by cancer and non-cancer patients, both of whom may benefit from a palliative approach to their care [30, 31]. Traditional medical practice tends to focus almost exclusively on curing illness and prolonging life while treating advanced illnesses, rather than on improving QoL and relieving suffering [32]. Contemporary trends in the treatment of heart failure advocate the integration of palliative care into comprehensive heart failure management [33, 34]. Regarding the extensive healthcare concerns raised during end-stage HF, recent literature and guidelines indicate that the provision of palliative care (PC) for end-stage HF patients is important. The American College of Cardiology and American Heart Association Guidelines [35], the Heart Failure Association of the European Society for Cardiology [36], the Canadian Cardiovascular Society [37], and the Hong Kong College of Physicians [38] all state that a palliative approach to care for patients with end-stage HF should be adopted.

Palliative care has a long history among patients with cancer, and the evidence in palliative care research is substantial [39]. In recent years, although it has become clear that palliative care can also benefit non-cancer patients, a systematic review looking at the effectiveness of specialized palliative care concluded that evidence of the benefits of this kind of care is sparse. Some authors have recommended carefully planned trials using a standardized palliative care intervention for terminally ill patients [40]. Specialized intervention [41] and coordination of care for patients with end-stage HF is needed to help reduce the risk of care fragmentation and to improve communication when many health professionals are involved [36].

Therefore, we designed the present study aiming to compare the effects of customary hospital-based care and an interventional Home-based Palliative HF Program (HPHP). Our objective is to examine the effect of the HPHP on hospital readmission rate, quality of life, functional status, symptom intensity, and satisfaction with care. The key outcome of this study is the hospital readmission rate, and the primary endpoint is 4 weeks post-discharge. The hypothesis is that patients with end-stage heart failure (ESHF) receiving the HPHP will have a lower hospital readmission rate than those receiving customary hospital-based care at 4 weeks post-discharge.

## Methods/design

### Overview of study design

This is a two-group, single-blinded randomized controlled trial. Participants are randomly allocated to two groups: the customary hospital-based (control) group and the HPHP (intervention) group. Eligible participants will be recruited from three regional hospitals in Hong Kong. The Standard Protocol Items: Recommendations for Interventional Trials (SPIRIT) guidelines for the trial are presented in Additional file 1.

### Preparatory phase

The preparatory phase includes the development of evidenced-based intervention protocols (6 months) and a training program for service providers (6 months).

### Trial phase

**Fig. 1:**
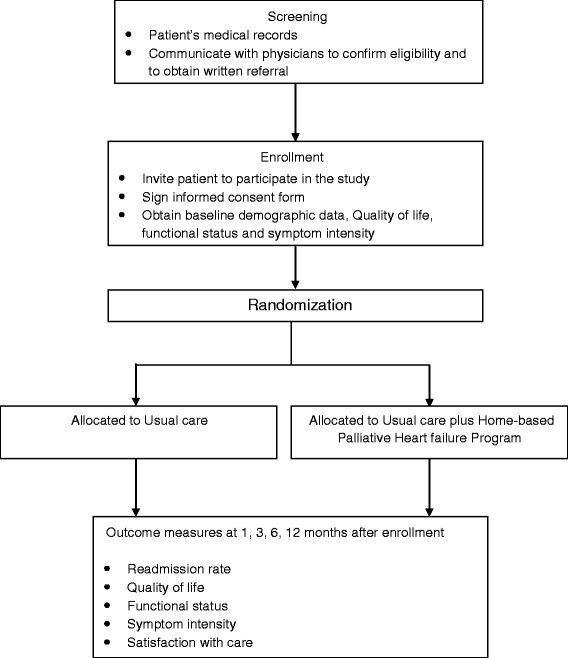
Flowchart for transition palliative care trial

This study (that is, the baseline assessment of subjects and intervention implementation for up to 12 months) will take 12 months. Intervention effects will be measured at 1 month, 3 months, 6 months, and 12 months and compared with the baseline measurements, as well as between the HPHP and control groups (Fig. 1).

### Conceptual framework

Studies have shown that supportive care programs using a case management approach can reduce readmission rates [42] and enhance quality of life [43]. Results from analyzing 96 sets of case notes of home visits to hospice patients showed that pain and edema can be quite well managed through palliative home care services, but breathlessness less so. Patients also experienced a number of social and psychological issues, such as role change, guilt, and anger, and palliative care proved helpful in reducing the intensity of these symptoms [29].

This intervention introduces a comprehensive Home-based Palliative HF Program (HPHP) to post-discharge patients with ESHF. The HPHP is built on two main conceptual guides: the recommended six principles for palliative HF care [44] and the four-Cs transitional care model. The six principles are as follows: 1) there is case management with coordination of care, communication and periodic review of treatment goals, and a symptom control plan with patient and family; 2) there are opportunities to discuss end-of-life issues; 3) a multidisciplinary approach is adopted; 4) there is a need for staff development, including the art of communication, for cardiovascular and palliative care; 5) topics for discussion of end-of-life issues include advanced directives, preference for treatment plan, and periodic re-evaluation of preference and treatment goals; 6) resources are provided that support an integrated model of HF palliative care [44].

A transitional care model embracing the preceding six principles will be adopted to guide the intervention design of this study. This study took reference of the transitional care model structure built by Naylor [45] and Coleman [46], which includes interfacing the pre-discharge phase with the post-discharge phase, continued with planned, proactive, and sustained follow-up for the post-discharged patients [42]. The design of the transitional care model in this study shares the essence of transitional care proposed by Naylor and Coleman to ensure post-discharge healthcare continuity and to avoid preventable readmissions [39, 45–47] but differs in the content by introducing the principles of HF palliative care as mentioned above. The key features in the transitional model of care to be used in this study are known as the four Cs, and were proposed by Wong et al. [42, 43, 48–50]. The four Cs refer to comprehensiveness, continuity, coordination, and collaboration. Being comprehensive means that the multidisciplinary team, together with the case manager, will conduct a holistic assessment of a patient’s condition and assume responsibility for anticipating the patient’s needs and facilitating the transition to home. Continuity of care is ensured by timely, proactive, and sustained follow-up on a regular basis. Coordination refers to the nurse case manager facilitating the communication with physicians, social workers, and other healthcare team members to respond to patients’ needs when necessary. Collaboration occurs not only among healthcare team members, but also between the provider and the patient/family. The patient/family needs to be involved as a partner, an active agent of care [42, 50].

### Study settings and participants

This is a multisite study conducted in collaboration with the palliative care teams from three regional hospitals. In-hospital patients with ESHF are reviewed by the physicians, and patients who are seen to be not benefiting from active medical treatment are considered for palliative care. The general inclusion criteria for subject recruitment include the ability to speak Cantonese, living within the hospital service area, and the ability to be contacted by phone. Specific inclusion criteria are based on the National Gold Standards Framework, Prognostic Indicator Guidance 2008 [51], in which patients must fulfill at least two of the indicators: (i) HF with New York Heart Association stage III or IV, (ii) patient thought to be in the last year of life, (iii) repeated hospital admissions with symptoms of HF, (iv) existence of physical or psychological symptoms despite optimal therapy. The nurse who is responsible for recruitment identifies eligible subjects, and the physician confirms that the patient fulfills the specific criteria and refers him/her to the PC team. Those with cognitive impairment, diagnosed with severe psychiatric disorders in their medical record, or discharged to a nursing home or other institution are excluded.

### Recruitment procedures

The ESHF patients who fulfill the set criteria are placed on the palliative care pathway. The research team approaches the patients for subject recruitment. A written consent form is signed if patients agree to participate in the study. After obtaining written informed consent, a research assistant who is blinded to the treatment assignment conducts a baseline interview, including participants’ demographic data. The self-rated measures adopted in this study are detailed in the outcome measures section below.

### Randomization and blinding

Randomization in permuted blocks is used. The permuted block randomization scheme was generated by computer software at www.randomization.com [52]. Randomization is done by an independent research team member who is not involved in any other parts of the study, in order to guarantee allocation concealment. The group assignment is placed in a sealed envelope, which is retained by the site investigator, who has no connection with the clinical team. Having successfully recruited a subject, the research nurse calls the independent site investigator to open an envelope and assign a group code to the participant according to the randomization scheme. The independent site investigator is blinded to the meaning of the group code. The participants and family members need to be introduced to the intervention program and they are not blinded. The intervention nurses are also not blinded since they need to deliver the intervention. Only the research assistants who help to collect the data are blinded to the group assignment.

### Study intervention

#### Usual care

Participants in both groups receive customary hospital-based care, which is a palliative care consultation service. The service includes communication on diagnosis, prognosis, advance care planning, symptom assessment, and caregiver support by a palliative physician or nurse. Home visits and telephone calls may be provided as part of the customary service if deemed necessary, and the number of visits and calls provided will be recorded for reference in the final analysis.

#### Usual care plus HPHP

In this group, the Home-based Palliative HF Program (HPHP) is initiated and supported by a core multidisciplinary palliative care team. The composition of the core team includes a palliative care nurse who acts as a nurse case manager (NCM), a palliative specialist, a social worker, and a group of trained nursing students as volunteers. The effect of using volunteers adds value, as shown in a local study [42] and elsewhere [53, 54]. The HPHP was developed based on the six principles for palliative care and the four-Cs transitional model, and as described in the conceptual framework section. Structured and regular home visits and telephone calls are two common approaches that can bring about positive effects in transitional care [23, 42, 50, 55, 56]. The comprehensive package of care will be guided by evidence-driven protocols formulated by the research team.

Below are the descriptions of the protocols.

1. Protocol for home visits – The first visit will be jointly delivered by the NCM and nursing students. Subsequent monthly visits will be conducted by the NCM or nursing students separately. The NCM will follow up on the health problems identified in the pre-discharge assessment in the first home visit. The subsequent home visits will follow up on the issues and goals of the previous visit. The nursing students will focus on social issues and report to the NCM and social worker after each home visit. The NCM will help set mutual goals with the patient to enhance optimal symptom control as well as social, psychological, and spiritual well-being. The NCM will consult the physicians in palliative care for clinical decisions, if needed. All home visits will be documented on structured forms designed based on the Omaha System [57]. The Omaha System was originally used in the United States. In Hong Kong, the research team has used it and found it comprehensive and valid for use with community-dwelling patients, including medical and hospice groups [29, 42, 49].
2. Protocol for telephone follow-up – The NCM will initiate calls between home visits to monitor progress, provide health advice, reinforce appropriate health behavior, assess the need for referral, and review management goals with the patients. The Omaha System will continue to be used as a framework to guide practice and documentation.
3. Protocol for referral system – A referral protocol will be established in case patients’ needs require further help, including: (a) social services – social, psychological, and financial support; (b) pastoral service – spiritual support; (c) medical clinic – medical review, drug adjustment; (d) nurse clinic – health assessment, non-pharmacological measures of symptom control; (e) emergency room – urgent conditions.
4. Protocol for patient-initiated calls – Besides provider-initiated home visits and telephone calls, patients can initiate calls to the NCM if they require advice and seek help before the next structured activity. The NCM will manage the calls according to the telephone follow-up and referral protocols. The content and duration of the calls will be documented.

### Training program for nurse case managers and volunteers

#### Training nurse case managers

The NCMs already have 6 years of clinical experience in palliative and/or HF care and have completed the post-registration specialty course. They have participated in a training program to enable them to master the key elements of the HPHP and enhance their relevant knowledge and skills (see Additional file 2). The training program was delivered by the research team in partnership with experienced clinical experts.

### Training volunteers

Volunteers are final year nursing students from a local nursing school, who have completed a 9-hour training program including theory- and practice-based content [39]. The training program was delivered by the research team.

### Pre-discharge phase

#### Assessment

Before the patient is discharged, the NCM will conduct a pre-discharge interview with the patient and family. The NCM will discuss the patient’s current physical, psychological, social, and spiritual status with the patient and family, as well as their preferred care approach. The patient and family will be encouraged to ask questions and to express their views throughout the process. At the end of the interview, the NCM will convene to develop a care plan in accordance with the wishes of the patient and family. Upon patient discharge, the nurse will meet with the patient and family to explain the HPHP arrangements and to document the assessment and plan using the Omaha System, which encompasses a comprehensive assessment, intervention, and evaluation scheme [57].

### Post-discharge phase

The first month post-discharge will include weekly care support: week 1 - the NCM and nursing students conduct a home visit together; week 2 - the NCM makes a telephone follow-up call; week 3 - the nursing students conduct a home visit in pairs; week 4 - the NCM makes a telephone follow-up call.

### Second and subsequent months post-discharge

The NCM will provide a monthly home visit, supplemented by a social visit and a telephone follow-up by nursing students each month. The patient will receive the HPHP for up to 12 months.

### Outcome measures

Data for the outcome measures will be collected at five time points: at discharge and at 1, 3, 6, and 12 months post-discharge. A research assistant with no clinical association with the patients will be responsible for collecting the data. In order to ensure the quality of the data collected, the research assistant will be trained in using different data collection tools. A value of 10 % will be used to test the intra-rater reliability, which involves the assistant collecting the same set of data twice within a short interval of time. All the data is independently checked by a member not involved in data collection to ensure the quality of the data collected. All data will be stored in a locked cabinet and will be entered in encrypted files to ensure data protection.

### Primary outcome

The primary outcome is quality of life, which will be measured by an HF-specific scale, the Chronic Heart Failure Questionnaire - Chinese version (CHQ) at 4 weeks post-discharge. The CHQ is one of the most commonly used HF-specific QOL instruments. It was developed by Guyatt et al. [58] in Canada. The tool has 20 items on a 7-point Likert scale measuring four domains: dyspnea, fatigue, emotional status, and mastery. The CHQ has been translated into Chinese and validated [59]. The internal consistency and test-retest reliability are good, with a Cronbach’s alpha of 0.95 and an intra-class correlation coefficient (ICC) of 0.75 [59]. In addition, the quality of life will also be measured by the McGill Quality of Life Questionnaire - Hong Kong version (MQOL-HK), which is a palliative-specific instrument measuring four domains: physical, psychological, existential, and support on a numerical scale from 0 to 10. It has been validated among palliative patients in Hong Kong with good reliability (the ICC is 0.85 (*p* < 0.0001) and the Cronbach’s alpha is 0.83) [60].

### Secondary outcomes

Evaluated health outcomes will be functional status, symptom intensity, and satisfaction with care. Functional status will be measured by the Palliative Performance Scale (PPS). The PPS, developed by Anderson et al. [61] based on the Karnofsky Performance Scale, is a tool specifically designed for palliative patients that reflects the changing physical condition in five aspects: ambulation, activities/evidence of disease, self-care, intake, and level of consciousness. The PPS is a clinical tool commonly used in local settings. The level of physical performance is rated on a scale of 100 (normal) to 0 (death), measured in 10 % decrement levels. The scale has been validated and the inter-rater reliability between doctors and nurses was maintained at .85 with strong kappa values of .97 [62]. The internal consistency is also good, with a Cronbach’s alpha equal to 0.83 [63].

The symptom intensity will be measured by the Edmonton Symptom Assessment System (ESAS). The ESAS assesses nine symptoms: pain, fatigue, nausea, depression, anxiety, drowsiness, appetite, sensation of well-being, and dyspnea, measured on a 0–100 mm visual analog scale (VAS). One extra empty VAS is left for the assessment of a less frequent symptom that may be important for an individual patient. The ESAS is widely adopted locally, and has been found to be a valid, reliable instrument for use among palliative care patients [64, 65]. A local study has also shown that the ESAS can help quantify symptoms and is an independent prognosticator for survival [66].

Satisfaction with care will be measured by the 15-item questionnaire used in the study of Wong et al., with validity confirmed by an expert panel and a reported test-retest reliability of .87 [42].

The service utilization outcome is measured by the hospital readmission rate, length of stay, and emergency room visits. The information will be extracted from the hospital administrative systems.

### Statistical methods

#### Sample size estimation

We estimated the sample size based on the study by Brannstrom and Boman [67], which detected a significant improvement in quality of life (intervention 60.4 versus control 52.3) after the structured palliative home care intervention provided by a multidisciplinary team among heart failure patients. A sample size of 117 for each arm is required to achieve a power of 80 % at a significance level of 0.05. To account for a 20 % attrition rate, 140 subjects for each arm with a total of 280 subjects are needed.

### Data analysis

We will use SPSS 22.0 for Windows to perform a statistical analysis. Descriptive statistics and means will be used to describe the participants’ background information and clinical variables. Poisson regression will be used to examine the mean difference for the hospital readmission rate between groups. A generalized estimating equation (GEE) will be used to examine the group, time, and interaction effects on the secondary outcome variables. The GEE is chosen because it treats observations of longitudinal data as correlated and thus is more robust in estimating standard errors [68].

The final report will follow the Consolidated Standards of Reporting Trials (CONSORT) statement as well as its extension for non-pharmacological interventions/complex intervention.

### Ethical considerations

This study has been reviewed and approved by the Research Ethics Committees of the two clusters in which the three hospitals are situated, the Institutional Review Board of the University of Hong Kong/Hospital Authority Hong Kong West Cluster, and the Kowloon Central/Kowloon East Cluster Research Ethics Committee (HKU/HA HKW IRB UW12-202; KC/KE120062/ER2), as well as by the Human Subjects Ethics Sub-Committee of the Hong Kong Polytechnic University (HSEARS20111003010).

## Discussion

Although there is evidence that successful transitional care interventions are effective in reducing readmission rates [46, 47, 69], the subjects in previous studies were chronically ill or elderly persons and there was no palliative care element involved. A recent systematic review conducted to examine the effect of transitional care interventions in preventing readmission for patients with heart failure revealed that patients with end-stage cardiovascular diseases were commonly not included in the trial; therefore, the results may not be applicable to the severely ill population [23]. Although randomized controlled trials of home-based programs had previously been conducted on severely ill patients [4, 70, 71], the interventions were initiated by health maintenance organizations [4, 70] and in an outpatient setting [71], while the intervention in our study begins in the in-patient hospital setting, aiming to ensure continuity of care in the hospital-to-home interface. In addition, Hansen and his colleagues [72] have evaluated interventions to reduce readmission within 30 days of hospital discharge, concluding that they did not identify distinct interventions or multicomponent discharge bundles that appeared to reliably decrease hospital readmission rates. Recent studies examined the impact of palliative care interventions on readmission rates and found that palliative care post-discharge could reduce 30-day hospital readmissions among seriously ill patients [73, 74]. However, these were retrospective studies. Other palliative care interventions were targeted at a group with late-stage diseases including cancer, chronic obstructive pulmonary disease (COPD), and end-stage renal disease [75, 76], but did not test any patients from the end-stage heart failure population. Few randomized controlled trials have been conducted to inform practitioners which models work best for end-stage HF patients under palliative care. In one of them, Brumley [4] compared the effects of usual care with a home-based palliative care intervention delivered by an interdisciplinary team to a group of terminal patients with HF, COPD, and cancer, reporting that those enrolled in palliative care made significantly less use of the emergency department (palliative 20 % versus usual care 33 %) and hospitalization (palliative 36 % versus usual care 59 %), resulting in an average 45 % decrease in costs as compared to patients under usual care. Outcome measures other than service use and the cost of the effects of palliative care have been reported. Morrison [77] translated the palliative care service into costs and found that the palliative group experienced significant reductions in costs involving admissions, laboratory tests, pharmacy services, and intensive care services compared to the usual care group.

The value of this study is threefold: to the healthcare system, to the patients, and to the science of healthcare research. If the HPHP proves successful, it could help reduce the burden on over-taxed hospital services by building a transitional care model that gradually shifts care from the hospital to the community. The second benefit of this project is for patients. The HPHP aims at helping patients with end-stage HF to control symptoms and maintain quality of life using a home-based care model. The patients can then remain in an environment that is familiar to them, receiving support from the healthcare team after hospital discharge. The third aspect of value is the contribution of this study to the science of healthcare research. Conventional end-of-life research tends to focus on single aspects, such as advanced directives, and targets mainly the cancer group. This study is an original effort to design a multidisciplinary transitional care model for end-stage HF patients and to subject it to scientific testing using a randomized controlled trial. The results of this study will help to inform practitioners and policy makers in planning and developing effective models that will fill the service gap and enhance quality of care for non-cancer patients at the end of life.

## Trial status

This study is currently at the final stage of data collection.

## Additional files

Additional file 1: SPIRIT Checklist. (DOCX 58 kb) Additional file 2: Training program for nurse case managers. (DOC 45 kb)

## Abbreviations

CHQ: Chronic Heart Failure Questionnaire
HF: heart failure
HPHP: Home-based Palliative Heart Failure Program
NCM: nurse case manager
PPS: Palliative Performance Scale
QoL: quality of life
PC: palliative care

## References

[1] Adler ED, Goldfinger JZ, Kalman J, Park ME, Meier DS (2009). Palliative care in the treatment of advanced heart failure. Circulation.

[2] Remme WJ, McMurray JJ, Rauch B, Zannad F, Keukelaar K, Cohen-Solal A (2005). Public awareness of heart failure in Europe: first results from SHAPE. Eur Heart J.

[3] Hung YT, Cheung NT, Ip S, Fung H (2000). Epidemiology of heart failure in Hong Kong, 1997. Hong Kong Med J.

[4] Brumley R, Enguidanos S, Jamison P, Seltz R, Norgenstem N, Saito S (2007). Increased satisfaction with care and lower costs: results of a randomized trial of in‐home palliative care. J Am Geriatr Soc.

[5] Centre for Health Protection, D.o.H. Number of deaths by leading causes of death 2001-2010. 2011. http://www.chp.gov.hk/en/data/4/10/27/117.html. Accessed 01 Jan 2010.

[6] Blinderman CD, Homel P, Billings JA, Portenoy RK, Tennstedt SL (2008). Symptom distress and quality of life in patients with advanced congestive heart failure. J Pain Symptom Manag.

[7] Foundation BH (2002). Mortality from heart failure.

[8] Selman L, Harding R, Beynon T, Hodson F, Hazeldine C, Coady E (2007). Modelling services to meet the palliative care needs of chronic heart failure patients and their families: current practice in the UK. Palliative Med.

[9] Leung KC, Leung KP, Chan KM, Tang SW. Prevent Rehospitalisation Program for High Risk Heart Failure Patients. J Hong Kong College Cardiol. 2008;16 Suppl p. 1.

[10] McMurray JJ, Stewart S (2002). The burden of heart failure. Eur Heart J Suppl.

[11] Jeon Y-H, Kraus SG, Jowsey T, Glasgow NJ (2010). The experience of living with chronic heart failure: a narrative review of qualitative studies. BMC Health Serv Res.

[12] Zambroski CH, Moser DK, Bhat G, Ziegler C (2005). Impact of symptom prevalence and symptom burden on quality of life in patients with heart failure. Eur J Cardiovasc Nur.

[13] Hägglund L, Boman K, Lundman B (2008). The experience of fatigue among elderly women with chronic heart failure. Eur J Cardiovasc Nur.

[14] Horne G, Payne S (2004). Removing the boundaries: palliative care for patients with heart failure. Palliative Med.

[15] Brännström M, Forssell A, Pettersson B (2011). Physicians’ experiences of palliative care for heart failure patients. Eur J Cardiovasc Nur.

[16] Ryan M, Farrelly M (2009). Living with an unfixable heart: a qualitative study exploring the experience of living with advanced heart failure. Eur J Cardiovasc Nur.

[17] Zambroski CH (2003). Qualitative analysis of living with heart failure. Heart Lung J Acute Critical Care.

[18] Nordgren L, Asp M, Fagerberg I (2007). Living with moderate-severe chronic heart failure as a middle-aged person. Qual Health Res.

[19] Aldred H, Gott M, Gariballa S (2005). Advanced heart failure: impact on older patients and informal carers. J Adv Nurs.

[20] Rhodes DL, Bowles CL (2002). Heart failure and its impact on older women’s lives. J Adv Nurs.

[21] Brännström M, Ekman I, Norberg A, Boman K, Strandberg G (2006). Living with severe chronic heart failure in palliative advanced home care. Eur J Cardiovasc Nur.

[22] Gott M, Small N, Barmes S, Payne S, Seamark D (2008). Older people’s views of a good death in heart failure: implications for palliative care provision. Soc Sci Med.

[23] Feltner C, Jones CD, Cené CW, Zheng ZJ, Sueta CA, Coker-Schwimmer EJ (2014). Transitional care interventions to prevent readmissions for persons with heart failure: a systematic review and meta-analysis. Ann Intern Med.

[24] Stamp KD, Machado MA, Allen NA (2014). Transitional care programs improve outcomes for heart failure patients: an integrative review. J Cardiovasc Nurs.

[25] Retrum JH, Boggs J, Hersh A, Wright L, Main DS, Magid DJ (2013). Patient-identified factors related to heart failure readmissions. Cir Cardiovasc Qual Outcomes.

[26] McMillan SC, Dunbar SB, Zhang W (2007). The prevalence of symptoms in hospice patients with end-stage heart disease. J Hosp Palliat Nurs.

[27] Johnson M, Houghton T (2006). Palliative care for patients with heart failure: description of a service. Palliative Med.

[28] Tse DMW, Chan KS, Lam WM, Leu KS, Lam PT (2007). The impact of palliative care on cancer deaths in Hong Kong: a retrospective study of 494 cancer deaths. Palliative Med.

[29] Wong FKY, Liu CF, Szeto Y, Sham M, Chan T (2004). Health problems encountered by dying patients receiving palliative home care until death. Cancer Nurs.

[30] Solano JP, Gomes B, Higginson IJ (2006). A comparison of symptom prevalence in far advanced cancer, AIDS, heart disease, chronic obstructive pulmonary disease and renal disease. J Pain Symptom Manag.

[31] World Health Organisation. World Health Organisation Global Burden of Disease Report. 2008 http://www.who.int/healthinfo/global_burden_disease/2004_report_update/en/. Accessed 31 Mar 2011.

[32] Morrison RS, Meier DE (2004). Palliative care. New Engl J Med.

[33] Goodlin SJ, Hauptman PJ, Arnold R, Grady K, Hershberger RE, Kutner J (2004). Consensus statement: palliative and supportive care in advanced heart failure. J Card Fail.

[34] Goodlin SJ (2009). Palliative care in congestive heart failure. J Am Coll Cardiol.

[35] Hunt SA, Abraham WT, Chin MH, Feldman AM, Francis GS, Ganiats TG (2009). Focused update incorporated into the ACC/AHA 2005 guidelines for the diagnosis and management of heart failure in adults: a report of the American College of Cardiology Foundation/American Heart Association Task Force on Practice Guidelines developed in collaboration with the International Society for Heart and Lung Transplantation. J Am Coll Cardiol.

[36] Jaarsma T, Beattie JM, Ryder M, Rutten FH, McDonagh T, Mohacsi P (2009). Palliative care in heart failure: a position statement from the palliative care workshop of the Heart Failure Association of the European Society of Cardiology. Eur J Heart Fail.

[37] Arnold JMO, Liu P, Demers C, Dorian P, Giannetti N, Haddad H (2006). Canadian Cardiovascular Society consensus conference recommendations on heart failure 2006: diagnosis and management. Can J Cardiol.

[38] Hong Kong College of Physicians. A Position Paper of the Hong Kong College of Physician. Palliative Care: Setting the scene for the future. 2008. http://www.hkcp.org/docs/News/Position%20paper%20in%20Palliative%20Medicine.pdf. Accessed 02 Jan 2010.

[39] Lorenz KA, Lynn J, Dy SM, Shugaman LR, Wilkinson A, Mularski RA (2008). Evidence for improving palliative care at the end of life: a systematic review. Ann Intern Med.

[40] Zimmermann C, Riechelmann R, Krzyzanowska M, Rodin G, Tannock I (2008). Effectiveness of specialized palliative care: a systematic review. JAMA.

[41] LeMond L, Allen LA (2001). Palliative care and hospice in advanced heart failure. Prog Cardiovasc Dis.

[42] Wong FKY, Ho MM, Yeung SY, Tam SK, Chow SK (2011). Effects of a health-social partnership transitional program on hospital readmission: a randomized controlled trial. Soc Sci Med.

[43] Wong FKY, Chow SKY, Chan TMF (2010). Evaluation of a nurse-led disease management programme for chronic kidney disease: a randomized controlled trial. Int J Nurs Stud.

[44] Howlett JG (2011). Palliative care in heart failure: addressing the largest care gap. Curr Opin Cardiol.

[45] Naylor MD, Aiken LH, Kurtzman ET, Olds DM, Hirschman KB (2011). The importance of transitional care in achieving health reform. Health Aff.

[46] Coleman EA, Parry C, Chalmers S, Min SJ (2006). The care transitions intervention: results of a randomized controlled trial. Arch Intern Med.

[47] Naylor MD, Brooten DA, Campbell RL, Maislin G, McCauley KM, Schwartz JS (2004). Transitional care of older adults hospitalized with heart failure: a randomized, controlled trial. J Am Geriatr Soc.

[48] Wong FKY, Mok MPH, Chan T, Tsang MW (2005). Nurse follow‐up of patients with diabetes: randomized controlled trial. J Adv Nurs.

[49] Wong FKY, Chow S, Chung L, Chang K, Chan T, Lee WM (2008). Can home visits help reduce hospital readmissions? Randomized controlled trial. J Adv Nurs.

[50] Wong FKY, Chow SKY, Chan TMF, Tam SKF (2014). Comparison of effects between home visits with telephone calls and telephone calls only for transitional discharge support: a randomised controlled trial. Age Ageing.

[51] National Gold Standards Framework Centre. Prognostic Indicator Guidance 2011. http://www.goldstandardsframework.org.uk/cd-content/uploads/files/General%20Files/Prognostic%20Indicator%20Guidance%20October%202011.pdf. Accessed 01 Apr 2011.

[52] Randomization.com. http://www.randomization.com. Accessed 11 Sept 2011.

[53] Faulkner M, Davies S (2005). Social support in the healthcare setting: the role of volunteers. Health Soc Care Comm.

[54] Candy B, France R, Low J, Sampson L (2015). Does involving volunteers in the provision of palliative care make a difference to patient and family wellbeing? A systematic review of quantitative and qualitative evidence. Int J Nurs Stud.

[55] Wong FKY, Chau J, So C, Tam SKF, McGhee S (2012). Cost-effectiveness of a health-social partnership transitional program for post-discharge medical patients. BMC Health Serv Res.

[56] Wong FKY, So C, Chau J, Law AKP, Tam SKF, McGhee S (2014). Economic evaluation of the differential benefits of home visits with telephone calls and telephone calls only in transitional discharge support. Age Ageing.

[57] Martin KS (2005). The Omaha System: A key to practice, documentation, and information management.

[58] Guyatt GH, Nogradi S, Halcrow S, Singer J, Sullivan MJJ, Fallen L (1989). Development and testing of a new measure of health status for clinical trials in heart failure. J Gen Intern Med.

[59] Lee DTF, Yu DSF, Woo J (2005). Validation of the chronic heart failure questionnaire (Chinese version). Qual Life Res.

[60] Lo RS, Woo J, Zhoc KC, Li CY, Yeo W, Johnson P (2001). Cross-cultural validation of the McGill quality of life questionnaire in Hong Kong Chinese. Palliative Med.

[61] Anderson F, Downing GM, Hill J, Casorso L, Lerch N (1995). Palliative performance scale (PPS): a new tool. J Palliative Care.

[62] Myers J, Gardiner K, Harris K, Lillien T, Bennett M, Chow E (2010). Evaluating correlation and interrater reliability for four performance scales in the palliative care setting. J Pain Symptom Manag.

[63] Brumley RD, Enguidanos S, Cherin DA (2003). Effectiveness of a home-based palliative care program for end-of-life. J Palliat Med.

[64] Bruera E, Kuehn N, Miller MJ, Selmser P, Macmillan K (1991). The Edmonton Symptom Assessment System (ESAS): a simple method for the assessment of palliative care patients. J Palliat Care.

[65] Bakitas M, Lyons KD, Hegel MT, Balain S, Brokaw FC, Seville J (2009). Effects of a palliative care intervention on clinical outcomes in patients with advanced cancer: the Project ENABLE II randomized controlled trial. JAMA.

[66] Lam PT, Leung MW, Tse CY (2007). Identifying prognostic factors for survival in advanced cancer patients: a prospective study. Hong Kong Med J.

[67] Brannstrom M, Boman K (2014). Effects of person-centred and integrated chronic heart failure and palliative home care. PREFER: a randomized controlled study. Eur J Heart Fail.

[68] Hanley JA, Negassa A, Edwardes MD (2003). Statistical analysis of correlated data using generalized estimating equations: an orientation. Am J Epidemiol.

[69] Naylor MD, Brooten D, Campbell R, Jacobsen BS, Mezey MD, Pauly MV (1999). Comprehensive discharge planning and home follow-up of hospitalized elders: a randomized clinical trial. JAMA.

[70] Aiken LS, Butner J, Lockhart CA, Volk-Craft BE, Hamilton G, Williams FG (2006). Outcome evaluation of a randomized trial of the PhoenixCare intervention: program of case management and coordinated care for the seriously chronically ill. J Palliat Med.

[71] Hughes SL, Weaver FM, Giobbie-Hurder A, Manheim L, Henderson W, Kubal JD (2000). Department of Veterans Affairs Cooperative Study Group on Home-Based Primary Care, et al. Effectiveness of team-managed home-based primary care: a randomized multicenter trial. JAMA.

[72] Hansen LO, Young RS, Hinami K, Leung A, Williams MV (2011). Interventions to reduce 30-day rehospitalization: a systematic review. Ann Intern Med.

[73] Ranganathan A, Dougherty M, Waite D, Casarett D (2013). Can palliative home care reduce 30-day readmissions? Results of a propensity score matched cohort study. J Palliat Med.

[74] Enguidanos S, Vesper E, Lorenz K (2012). 30-day readmissions among seriously ill older adults. J Palliat Med.

[75] Gade G, Venohr I, Conner D, McGrady K, Beane J, Richardson RH (2008). Impact of an inpatient palliative care team: a randomized controlled trial. J Palliat Med.

[76] Lukas L, Foltz C, Paxton H (2013). Hospital outcomes for a home-based palliative medicine consulting service. J Palliat Med.

[77] Morrison RS, Penrod JD, Cassel B, Caust-Ellenbogen M, Litke A, Spragens L (2008). Cost savings associated with US hospital palliative care consultation programs. Arch Intern Med.

