# Supplementary material for: Effects of a transitional palliative care model on patients with end-stage heart failure: study protocol for a randomized controlled trial
Source: Trials. 2016 Mar 31;17:173. doi: 10.1186/s13063-016-1303-7 (PMC4815195; doi:10.1186/s13063-016-1303-7)
Supplement: Additional file 2: — Training program for nurse case managers. (DOC 45 kb) [file 13063_2016_1303_MOESM2_ESM.doc]

Online Supplement

Training program for the Nurse Case Managers (NCMs)

NCMs

The nurses had to have at least 3 years’ clinical experience in palliative and/or HF care and pass the exit competence test in writing and a practice case before the intervention.

Training Content (18 hours)

Part I (9 hours)

Section I (3 hours)

Concepts of transitional care model

Case management and approaches

Home visits and telephone calls

Role of the nurse case manager in the project

Section II (5 hours)

Holistic concerns for patients with end-stage heart failure

Protocols for symptom management

Available health and social resources

Integrated palliative HF care

Section III (2 hours)

Study flow and logistics

Communication among members of the service and research team

Documentation

Section III (8 hours)

Assessment, planning, intervention and documentation using the Omaha System

Practice with a training case

Case discussion
